# Large Cohort Screening of G6PD Deficiency and the Mutational Spectrum in the Dongguan District in Southern China

**DOI:** 10.1371/journal.pone.0120683

**Published:** 2015-03-16

**Authors:** Qi Peng, Siping Li, Keze Ma, Wenrui Li, Qiang Ma, Xiaoguang He, Yuejing He, Ting He, Xiaomei Lu

**Affiliations:** The Eighth People’s Hospital of Dongguan, Dongguan Institute of Pediatrics, Dongguan, Guangdong, China; Agency for Science, Technology and Research - Singapore Immunology Network, SINGAPORE

## Abstract

**Background:**

Glucose-6-phosphate dehydrogenase (G6PD) deficiency is a common enzymatic disorder of the erythrocytes that affects 400 million people worldwide. We developed a PCR-reverse dot blot (RDB) assay to screen twenty genotypes of seventeen Chinese G6PD mutations and investigate the spectrum of G6PD deficiency mutations in Dongguan District, Guangdong Province, in southern China.

**Method:**

The PCR-RDB assay consists of multiplex PCR amplification of seven fragments in the G6PD target sequence of wild-type and mutant genomic DNA samples followed by hybridization to a test strip containing allele-specific oligonucleotide probes. A total of 16,464 individuals were analyzed by a combination of phenotypic screening and genotypic detection using the PCR-RDB assay and DNA sequence analysis.

**Results:**

The PCR-RDB assay had a detection rate of 98.1%, which was validated by direct sequencing in a blind study with 100% concordance. The G6PD deficiency incidence rate in Dongguan District is 4.08%. Thirty-two genotypes from 469 individuals were found. The two most common variants were c.1376G>T and c.1388G>A, followed by c.95A>G, c.871G>A, c.392G>T, and c.1024 C>T. In addition, two rare mutations (c.703C>A and c.406C>T) were detected by DNA sequencing analysis. In our study, 65 cases harbored the C1311T/IVS polymorphism and 67 cases were homozygote.

**Conclusion:**

The PCR-RDB assay we established is a reliable and effective method for screening G6PD mutations in the Chinese population. Data on the spectrum of mutations in the Dongguan District is beneficial to the clinical diagnosis and prevention of G6PD deficiency.

## Introduction

Glucose-6-phosphate dehydrogenase (G6PD) deficiency is a multiethnic inherited disease with a particularly high prevalence in tropical and subtropical regions, including southern China [1]. The population frequencies of G6PD deficiency range from 3.1–16.1% in Guangdong Province, China [2–3]. G6PD deficiency is caused by X-linked, hereditary mutations in the *G6PD* gene, which result in protein variants with different levels of enzyme activity that are related to a wide range of biochemical and clinical phenotypes. Acute hemolysis, neonatal hyperbilirubinemia, and chronic hemolysis are the most common clinical manifestations in patients, which are triggered by exogenous agents, such as the ingestion of fava beans, certain drugs, infections, and metabolic conditions [4–5].

More than 180 mutations have been described worldwide [6]. There are at least 20 different point mutations that have been identified in the Chinese population; 13 point mutations (c.1376G>T, c.1388G>A, c.95A>G, c.871G>A, c.1004C>T, c.1004C>A, c.1024C>T, c.392G>T, c.1381G >A, c.835A>G, c.835A>T, and c.517T>C) and a silent polymorphism (c.1311C>T) account for more than 95% of all cases [3, 7–15]. The G6PD deficiency variants vary between different regions and ethnicities. However, very little is known about the spectrum of G6PD mutations in Dongguan District in the Guangdong Province of southern China. Dongguan is a prefecture-level city in central Guangdong and has an estimated 8.29-million inhabitants with a mean age of 30.82 years.

Currently, a variety of methods are used to detect G6PD mutations, such as polymerase chain reaction/restriction fragment length polymorphism (PCR-RFLP) and denaturing high-performance liquid chromatography (DHPLC). However, reverse dot blot (RDB) assays have been used to detect certain genetic disorders on the basis of technical simplicity and convenience.

In the present study, we aimed to establish an RDB assay to screen 20 genotypes of the 17 common G6PD mutations, including the thirteen most common mutations and seven adjacent mutations (c.487G>A, c.493A>G, c.519C>G, c.519C>T, c.592C>T, c.1360C>T, and c.1387C>A). By combining phenotype screening and genotype detection using a PCR-RDB assay and DNA sequence analysis, we have studied the incidence of G6PD deficiency and provide data on the spectrum of *G6PD* mutations in Dongguan. Our findings are critical to the development of a carrier screening and prevention program in the region's community.

## Materials and Methods

### Population

A total of 16,464 unrelated participants were enrolled in our study from January 2011 to December 2013 and included 12,579 females (mean age 27.86 ± 3.99 years) and 3,885 males (mean age 29.08 ± 4.08 years); The age ranged from newborn to 49 years. All of the study participants were local Han ethnic residents with Dongguan ancestry. Peripheral blood samples were randomly collected from these participants during routine physical examinations in our hospital. The study was approved by the Institutional Ethnics Committee of the Eighth People's Hospital of Dongguan. Informed written consent was obtained from all adult participants or the guardians of pediatric participants.

#### G6PD enzyme activity screening

Enzyme activity was measured using a commercial G6PD Detection Assay Kit (KOFA Medical), according to the method suggested by WHO for measuring the G6PD/6PGD ratio [16]. It is a simple and convenient screening method for G6PD deficiency, with a high specificity (up to 95%) and sensitivity of 87% [16]. All of the tests were conducted by following the manufacturer’s instructions. The reliability of the test results was monitored by calibration and by using controls provided by KOFA Medical in each test run. Adults with a G6PD/6PGD ratio below 1.00(normal range: 1.00~2.30) and infants with a ratio below 1.10 (normal range: 1.10~2.50) were considered to be G6PD deficient. We randomly selected 469 G6PD deficient samples for molecular analysis by the PCR-RDB assay.

### G6PD genotyping

#### Genomic DNA extraction

Genomic DNA extraction was performed using the QIAamp DNA Blood Mini kit (Qiagen, Hilden, Germany) according to the manufacturer's instructions; quantitative estimation of DNA was carried out by spectrophotometer (NanoDrop).

#### Primers and probes design

To amplify exons 2, 5, 6, 8, 9, 11, and 12 of the *G6PD* gene to include the 17 mutation sites found in China, we designed seven sets of primers for multiplex PCR (M-PCR) (Table 1). The 5′ ends of the primers were labeled with biotin. A total of 33 probes consisting of 13 normal and 20 mutant probes for the point mutation sites were designed (Table 2).

**Table 1:** Multiplex PCR primers.

Sequences of primer (5′–3′)		Mutations	Size (bp)
Exon 2	1F GTGTGAGACCCCAGAGGAAC	95	383
	1R TGCACACCAGGTAGAGCCG		
Exon 5	1F CCACCCCAGAGGAGAAG	392	294
	1R GACACGCTCATAGAGTGG		
Exon 6	1F CTCACTCCCCGAAGAGGGG	487,493,517,519,592	220
	1R CCCCACCTCAGCACCAT		
Exon 8	1F CACTAGGAAGCCTTGTTTGG	835	251
	1R TCAGTGCCTCGTCACAGAT		
Exon 9	1F CCACAGTCATCCCTGCAC	871,1004,1024	285
	1R TGCCTTGCTGGGCCTCG		
Exon 11	1F GGTGGCAGGCAGTGGCATCA	1311,1360	187
	1R ACAGGGAGGGAGGGCAAAGG		
Exon 12	1F CCTGCATACCTGTGGGC	1376,1381,1387,1388	195
	1R CCCCACCCTTTCCTCAC		

**Table 2:** G6PD gene mutation detection probes.

Name of probe	Mutation detection probe(5’→3’)	Name of probe	Mutation detection probe(5’→3’)
A95G	GGATACACGCATATTCATCA	95N	GGATACACACATATTCATCA
G392T	CTCCACCTGGTGTCACAG	392N	CTCCACCTGGGGTCACAG
G871A	AGGTCAAGATGTTGAAATG	871N	AGGTCAAGGTGTTGAAATG
C1004T	CACCACCGTCACTTTTGCA	1004N	CACCACCGCCACTTTTGCA
C1004A	CACCACCGACACTTTTGCA		
C1024T	AGCCGTCGTCTTCTATGT	1024N	AGCCGTCGTCCTCTATGT
C1360T	GCACTTCGTGTGCAGGTGA	1360N	GCACTTCGTGCGCAGGTGA
G1376T	GAGCTCCTTGAGGCCTGG	1376/1381N	GAGCTCCGTGAGGCCTGG
G1381A	GAGCTCCGTGAGACCTGG		
C1387A	CCTGGAGTATTTTCACCCC	1387/1388N	CCTGGCGTATTTTCACCCC
G1388A	CCTGGCATATTTTCACCCC		
G487A	GCTCCCAGCAGAAGCT	487/493N	CAGAGGCTGGAACCG
A493G	CTGGGACCGCATCAT		
T517C	CTCCCGAGGGGCTTC	517/519N	CTCCCGAAGGGCTTCTC
C519T	CTCCCAAAGGGCTTCTC		
C519G	CTCCCCAAGGGCTTCTC		
C592T	ATCTACTGCATCGACCA	592N	ATCTACCGCATCGAC
A835G	CGCCTCCGCCAACTCA	835N	CGCCTCCACCAACTCA
A835T	CGCCTCCTCCAACTCA		
C1311T	TGACGCCTATGAGCGC	1311N	TGACGCCTACGAGCG

6 of the normal probes were used as controls against 13 mutant probes: 1004N against c.1004 C>T and c.1004 C>A; 1376N against c.1376G>T and c.1381G>A; 1388N against c.1388G>A and c.1387C>T; 487N against c.487G>A and c.493G>A; 517N against c.517T>C, c.519C>T and c.519C>G; 835N against c.853A>G and c.85 A>T

### Amplification of M-PCR

M-PCR was performed using an Mx3000p PCR machine (Stratagene), with a final reaction volume of 50 μL containing 1 x PCR buffer (MgCl_2_ plus), 1 x Q buffer, 0.2 mmol/L of each dNTP (Promega), 0.2 μmol/L of each of the fourteen primers, 0.1 U/μL of HotStarTaq DNA Polymerase (Qiagen, Hilden, Germany) and 1 μg of genomic DNA. The manufacturer's suggested conditions were used for PCR: pre-denaturation at 96°C for 15 min; 35 cycles of denaturation at 95°C for 30 s, annealing at 55°C for 30 s, and extension at 72°C for 30 s; and a final extension at 72°C for 5 min. The PCR products were subsequently visualized by electrophoresis on a 1.5% agarose gel.

### RDB assay

**Fig 1:**
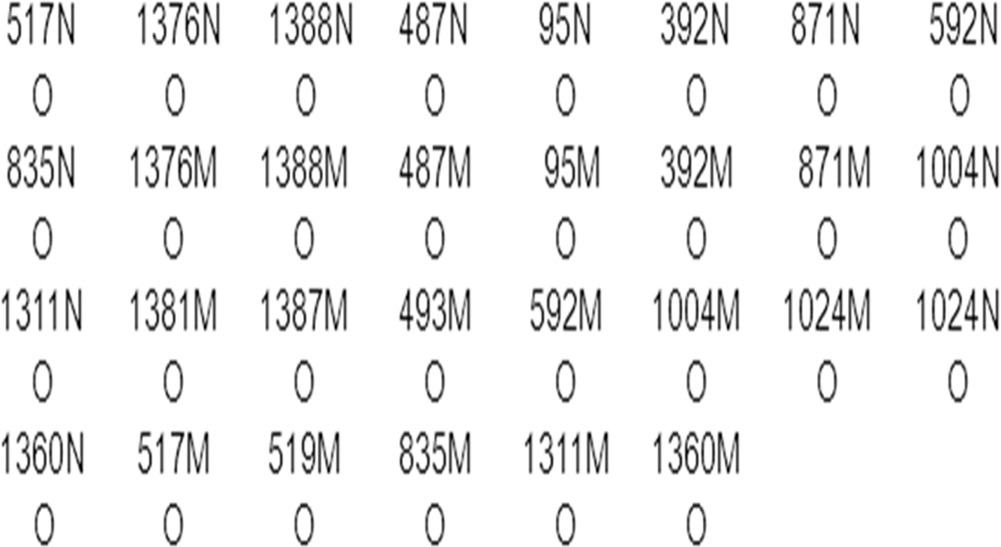
Layout of the probes’ dotting in the nylon strip designed for the reverse dot blot assay. N denotes the wild-type probes corresponding to each mutant probes (denoted at each mutation).

As shown in Fig. 1, all of the 33 probes were fixed to nylon strips (PALL Biodyne C). The amplified M-PCR products were placed in screw-top tubes with the strips and 8 mL of hybridization solution A (2 × SSC, 0.1% SDS, pH7.4) and were denatured at 100°C for 10 min and incubated at 45°C for 4 h. The strips were then removed to wash solution B (0.5 × SSC and 0.1% SDS, pH7.4) at 45°C for 10 min and were then transferred to a hybridization solution A diluted mixture containing 0.125 U/mL of streptavidin-POD conjugate (Roche, Mannheim, Germany) was incubated at room temperature for 30 min. Excess conjugate was removed with two additional washes using solution A. Finally, the color developing solution (pH 5.4, 0.1 mg/mL of TMB, 0.015‰ H_2_O_2_, and 0.1 mol/L sodium citrate) was applied. The color reaction lasted for 20 min, and positive results of detection appeared as blue dots.

### Sequence analysis

All samples found to be negative by the PCR-RDB assay were subjected to DNA sequencing of the whole coding region. One hundred positive samples from then PCR-RDB assay were randomly selected for validation by sequencing. PCR products were purified using the QIA quick PCR Purification Kit (Qiagen) and sequenced by Beijing Genomics Institution (Shenzhen).

## Results

### Establishment of the PCR-RDB assay

**Fig 2:**
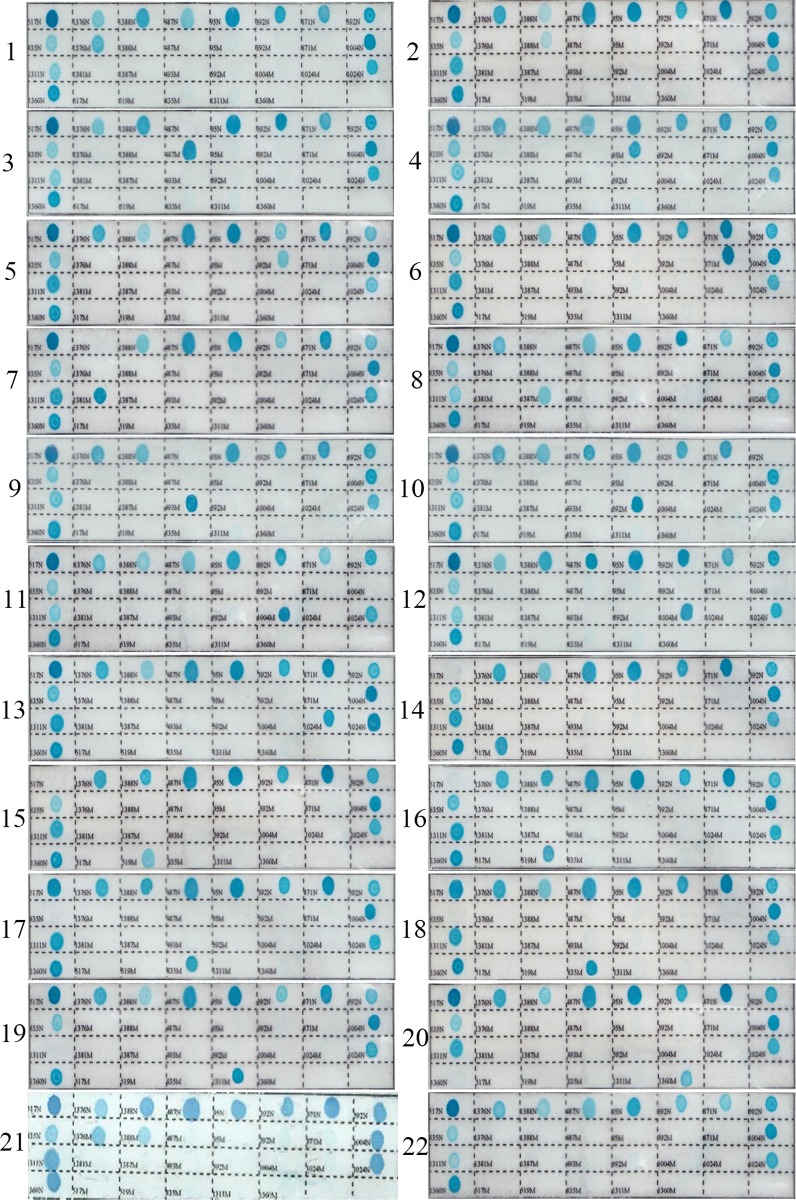
Representative genotyping results of G6PD deficiency using reverse dot blot assay. (1)c.1376 G>T heterozygotes;(2) c.1388 G>A heterozygotes; (3)c.487 G>A Hemizygote; (4)c.95 A>G heterozygotes; (5)c.392 G>T heterozygotes; (6)c.871 G>A heterozygotes; (7)c.1381 G>A Hemizygote; (8)c.1387 C>A Hemizygote; (9)c.493 A>G Hemizygote; (10)c.592 C>T Hemizygote; (11)c.1004 C>T Hemizygote; (12)c.1004 C>A Hemizygote; (13)c.1024 G>T heterozygotes; (14)c.517 T>C Hemizygote; (15)c.519 C>G Hemizygote; (16)c.519 C>T Hemizygote; (17)c.835 A>G Hemizygote; (18)c.835 A>T Hemizygote; (19)c.1311 C>T Hemizygote; (20)c.1360 C>T Hemizygote. (21)c.1376 G>T/c.1388 G>A double heter-ozygotes (22) normal control.

The PCR-RDB assay was established using DNA samples from nine male patients and the remaining eleven known mutations were obtained by synthetic gene sequencing (Beijing Genomics Institute). During the assay, color development of a normal spot served as the control for the color reaction system. Hemizygous DNA samples were indicated by blue spots for the appropriate mutant probes and white spots for the corresponding normal probes. Heterozygous and homozygous DNA samples were indicated by blue spots for one or two mutant probes, respectively, as well as blue spots for the corresponding control probes. Normal DNA samples had blue spots for all of the control probes. Because the G6PD gene is X-linked, gender was confirmed to match the number of alleles observed in each analysis. Representative results are shown in Fig. 2. DNA sequencing results of all 100 positive samples demonstrated 100% concordance with the PCR-RDB results.

### Population prevalence and mutation spectrum of G6PD deficiency in Dongguan District

Of the 16,464 samples that were screened, 672 were positive for G6PD deficiency, as assessed by the G6PD/6PGD ratios using the spectrophotometer method, and included 377 females and 295 males. Four hundred and sixty-nine G6PD-deficiency samples were randomly selected for molecular analysis via the PCR-RDB assay. Four hundred and sixty samples contained mutations, and the remaining nine were negative for all 17 of the assayed mutations. In the cohort that we investigated, 32 different genotypes were identified from 469 G6PD-deficient individuals (Table 3). In addition, we found 65 cases of the C1311T/IVS polymorphism, and 67 patients were homozygous for twelve different genotypes in our study (Table 3). All the male patients carrying c.871G>A, which is responsible for the G6PD Viangchan variant, had the C1311T/IVS polymorphism. The number and percentage of different deficient chromosomes observed in a subsample of “mainly” deficient male and female subjects are listed in Table 4. The total number of G6PD-deficient alleles in the males was 218, giving a frequency of 5.58% for G6PD deficiency in the Dongguan population. A total of 533 chromosomes with different known mutations were characterized in our study. The six most common variants in the Dongguan population were G6PD Canton (1376 G>T), G6PD Kaiping (1388 G>A), G6PD Gaohe (95 A>G), G6PD Viangchan (871 G>A), G6PD Chinese-4 (392 G>T), and G6PD Chinese-5 (1024 C>T).

**Table 3:** Thirty-two different genotypes associated with G6PD deficiency and their contributions in Dongguan.

Hemizygote	n (%)	Heterozygote	n (%)	Homozygote	n (%)
G1376T	62 (28.4)	G1376T	68^c^ (37.6)	G1376T	9 (13.4)
G1388A	79 (36.2)	G1388A	37^d^ (20.4)	G1388A	9 (13.4)
A95G	30 (13.8)	A95G	35^e^ (19.3)	A95G	7 (10.4)
G392T	11 (5.1)	G392T	7 (3.9)	G1376T/G1388A^h^	13(19.4)
C1024T	5 (2.3)	C1024T	5 (2.8)	G1376T/G871A	8(12.0)
G871A	22^a^ (10.1)	G871A	25^f^ (13.8)	G1376T/A95G	5(7.5)
T517C	2(0.9)	C1004T	2^g^ (1.1)	G1376T/C1024T	4(6.0)
C1004T	2^b^(0.9)	C703A	1 (0.6)	G1388A/A95G	3(4.5)
C1360T	1(0.5)	C401T	1 (0.6)	G1376T/G392T	2(3.0)
C703A	2(0.9)			G1376T/C1004T	2(3.0)
C406T	2(0.9)			G392T/G871A^i^	3(4.5)
				A95G/G871A^j^	2(3.0)
Total	218(100.0)		181 (100.0)		67(100.0)

a. All of the 22 individuals had the c.1311C>T silent polymorphism.

b. All of the 2 individuals had the c.1311C>T silent polymorphism.

c. Out of the 68 individuals, four subjects had the c.1311C>T silent polymorphism.

d. Out of the 37 individuals, two subjects had the c.1311C>T silent polymorphism.

e. Out of the 35 individuals, two subjects had the c.1311C>T silent polymorphism.

f. Out of the 25 individuals, twenty subjects had the c.1311C>T silent polymorphism.

g. All of the 2 individuals had the c.1311C>T silent polymorphism.

h. Out of the 13 individuals, seven subjects had the c.1311C>T silent polymorphism.

i. Out of the 3 individuals, two subjects had the c.1311C>T silent polymorphism.

j. All of the 2 individuals had the c.1311C>T silent polymorphism.

**Table 4:** The results of screening and genotyping 16 464 samples for G6PD deficiency in subsample of “mainly” deficient subjects in Dongguan District.

G6PD Variants	G6PD Mutations	Males		Females	
		n	%	n	%
Canton	G1376T	62	1.60	120	0.95
Kaiping	G1388A	79	2.03	71	0.56
Gaohe	A95G	30	0.77	59	0.47
Viangchan	G871A	22	0.57	38	0.30
Chinese-4	G392T	11	0.28	12	0.10
Chinese-5	C1024T	5	0.13	9	0.07
Nankang	T517C	2	0.05	-	-
Fushan	C1004T	2	0.05	4	0.03
Union	C1360T	1	0.03	-	-
Nanning	C703A	2	0.05	1	0.01
Valladolid	C406T	2	0.05	1	0.01
Total		218	5.58	315	2.50

The nine samples negative for the tested mutations by PCR-RDB were subjected to DNA sequencing of the whole coding region. The sequencing results revealed that three samples carried the c.703C>A mutation and another three carried c.406C>T. Three other samples were negative for all tested mutations.

## Discussion

G6PD deficiency is an X-linked inherited disorder that results from mutations in the *G6PD* gene [17]. Because the *G6PD* gene is located on the X chromosome, in males it occurs only as a normal (Gd+) or deficient (Gd-) hemizygous genotype, but in females, who have two copies of the *G6PD* gene on each X chromosome, it occurs as a normal homozygous (Gd+/Gd+), deficient homozygous (Gd-/Gd-) or heterozygous (Gd-/Gd+) genotype [17–18]. As a consequence, and due to lyonization (inactivation of one X chromosome), heterozygous women have two erythrocyte populations, each resulting from the expression of one of two G6PD alleles: one population may have normal or deficient G6PD levels, whereas the other population may have a different level of deficiency [4, 6]. Female heterozygotes with a defective G6PD variant present a different diagnostic challenge because their blood contains markedly varying proportions of normal and deficient cells [19]. Traditional screening procedures are robust in detecting the fully developed defect in males, but they fall short in detecting female heterozygotes and patients with relatively mild forms of G6PD deficiency, such as those with the A-variant who are experiencing hemolysis because the level of activity in young erythrocytes is higher than that in more mature cells [20–21]. Therefore, a cheap and reliable test is necessary for diagnosing the deficiency to prevent hemolytic disorders in developing countries.

The PCR-RDB assay is a well-developed method that can genotype multiple single nucleotide polymorphisms (SNPs) simultaneously and can be used for the diagnosis of homozygous, hemizygous, and heterozygous-deficient patients [22]. It has been successfully used for several genetic disorders, such as thalassemia and human papilloma virus typing [22–25]. In the present study, we successfully established an RDB assay for screening 20 common genotypes of the 17 common Chinese G6PD mutations. Of the 469 samples that screened positive for G6PD enzyme activity, 460 were genotyped via PCR-RDB assay. The detection rate was 98.1%, and the DNA sequencing results of 100 randomly selected genotyped samples showed 100% concordance. This method includes M-PCR amplification, hybridization, washing, and color development. It is a highly efficient and low-cost method, as it is easily performed with commonly available equipment and can be completed within 6 hours.

Molecular analyses have shown that different ethnic groups bear different characteristic profiles for G6PD-deficiency variants [26]. Our work aimed to evaluate the overall prevalence of G6PD deficiency in Dongguan District by screening and genotyping blood samples from 16,464 individuals. The G6PD deficiency incidence rate in the Dongguan area was consistent with the reported data from Guangdong Province [27]. The rate of the C1311T/IVS polymorphism in 469 individuals with G6PD deficiency was 13.9% (65 of 469), and all the 22 male patients with G6PD Viangchan had the C1311T/IVS polymorphism. The silent polymorphism at nucleotide position 1311 (C-T) in exon 11 does not result in any substitutions at the amino acid level; it was previously noted that there was similar linkage disequilibrium between mutation 871A and polymorphic site 1311 T in Asians [28–30].

Overall, the PCR-RDB assay for G6PD genotyping is an ideal method for smaller clinics and laboratories with limited resources that need to perform front-line testing without using direct DNA sequencing [15]. G6PD phenotypic screening combined genetic analyses can be used to identify hemizygous males and homozygous or heterozygous females for the prevention of acute hemolytic crisis; to identify heterozygous females, especially among high risk families; to evaluate neonatal jaundice in newborn boys; to recognize novel mutations in G6PD deficient cases; and to gather information for further family studies. Therefore, this screening protocol is capable of increasing the level of prevention of this disease [19].

## References

[1] Beutler E. Glucose-6-phosphate dehydrogenase deficiency: a historical perspective. Blood. 2008; 111:16–24. 18156501 10.1182/blood-2007-04-077412

[2] Xu YK, Zheng RP, Liu LB, Hua XY, Zhong JP, Zhu XP, et al Investigation of red cell glucose-6-phosphate dehydrogenase deficiency gene frequency in 9 nationality populations in 7 provinces (Autonomous Regions). Heredity Dis. 1985;2:67–71.

[3] Du CS, Xu YK, Hua XY, Wu QL, Liu LB. Glucose-6- phosphate dehydrogenase variants and their frequency in Guangdong, China. Hum Genet. 1998; 80:385–388. 10.1007/BF00273657 3198117

[4] Cappellini MD, Fiorelli G. Glucose-6-phosphate dehydrogenase deficiency. Lancet.2008;371:67–74. 10.1016/S0140-6736(08)60073-2 18177777

[5] Beutler E. G6PD deficiency. Blood. 1994; 84:3613–3636. 7949118

[6] Minucci A, Moradkhani K, Hwang MJ, Zuppi C, Giardina B, Capoluongo E. Glucose-6-phosphate dehydrogenase (G6PD) mutations database: review of the “old” and update of the new mutations. Blood Cells Mol Dis. 2012; 48:154–165. 10.1016/j.bcmd.2012.01.001 22293322

[7] Chiu DT, Zuo L, Chao L, Chen E, Louie E, Lubin B, et al Molecular characterization of glucose-6-phosphate dehydrogenase (G6PD) deficiency in patients of Chinese descent and identification of new base substitutions in the human G6PD gene. Blood. 1993; 81:2150–2154. 8471773

[8] Li L, Zhou YQ, Xiao QZ, Yan TZ, Xu XM. Development and evaluation of a reverse dot blot assay for the simultaneous detection of six common Chinese G6PD mutations and one polymorphism. Blood Cells Mol Dis. 2008; 41:17–21. 10.1016/j.bcmd.2008.01.007 18329300

[9] Pan SL. G6PD deficiency: distribution in East and Southern Asia and positive selection by malaria. Chin J Health Birth Child Care. 2007; 13:42–52.

[10] Jiang W, Yu G, Liu P, Geng Q, Chen L, Lin Q, et al Structure and function of glucose-6-phosphate dehydrogenase- deficient variants in Chinese population. Hum Genet. 2006; 119: 463–478. 16607506 10.1007/s00439-005-0126-5

[11] Yan JB, Xu HP, Xiong C, Ren ZR, Tian GL, Zeng F, et al Rapid and reliable detection of glucose-6-phosphate dehydrogenase (G6PD) gene mutations in Han Chinese using high-resolution melting analysis. J Mol Diagn. 2010; 12: 305–311. 10.2353/jmoldx.2010.090104 20203002 PMC2860466

[12] Yan T, Cai R, Mo O, Zhu D, Ouyang H, Huang L, et al Incidence and complete molecular characterization of glucose-6-phosphate dehydrogenase deficiency in the Guangxi Zhuang autonomous region of southern China: description of four novel mutations. Haematologica.2006; 91: 1321–1328. 17018380

[13] Chiang SH, Wu SJ, Wu KF, Hsiao KJ. Neonatal screening for glucose-6-phosphate dehydrogenase deficiency in Taiwan. Southeast Asian J Trop Med Public Health. 1999; 30 Suppl 2:72–74. 11400791

[14] Tang TK, Huang WY, Tang CJ, Hsu M, Cheng TA, Chen KH. Molecular basis of glucose-6-phosphate dehydrogenase (G6PD) deficiency in three Taiwan aboriginal tribes. Hum Genet. 1995; 95:630–632. 7789945 10.1007/BF00209477

[15] Lu X, Hua L, Zhang T, Li S, Fan X, Peng Q, et al A reverse dot blot assay for the expanded screening of eleven Chinese G6PD mutations. Clin Chim Acta. 2013;418:45–49. 10.1016/j.cca.2012.12.023 23313052

[16] Chen Y, Jiang W, Li C. A study on the influential factors of G6PD/6PGD specific value assay in the heterozygotes of G6PD gene variants in female patients. Int J Lab Med.2006; 27:385–390.

[17] Adam A. Linkage between deficiency of glucose-6- phosphate dehydrogenase and colour-blindness. Nature.1961; 189: 686 13681296 10.1038/189686a0

[18] Zaffanello M, Rugolotto S, Zamboni G, Gaudino R, Tatò L. Neonatal screening for glucose-6-phosphate dehydrogenase deficiency fails to detect heterozygote females. Eur J Epidemiol. 2004;19:255–257. 15117119 10.1023/b:ejep.0000020445.48298.3f

[19] Minucci A, Giardina B, Zuppi C, CapoluongoE. Glucose-6-phosphate dehydrogenase laboratory assay: How, when and why? IUBMB Life. 2009; 61:27–34. 10.1002/iub.137 18942156

[20] Herz F, Kaplan E, Scheye ES. Diagnosis of erythrocyte glucose-6-phosphate dehydrogenase deficiency in the negro male despite hemolytic crisis. Blood.1970; 35:90–93. 5412679

[21] Beutler E, Blume KG, Kaplan JC, Löhr GW, Ramot B, Valentine WN. International Committee for Standardization in Haematology: recommended screening test for glucose-6- phosphate dehydrogenase (G-6-PD) deficiency. Br J Haematol. 1979;43:465–467. 497122 10.1111/j.1365-2141.1979.tb03774.x

[22] Li Y, Chen Z, Zhao L, Wang L. Molecular diagnosis for a novel deletion mutation of α thalassemia. Zhonghua Xue Ye Xue Za Zhi.2014; 35:724–727. 10.3760/cma.j.issn.0253-2727.2014.08.012 25152121

[23] Hao Y, Xu ZY, Jin Q, Wu WQ. Molecular and prenatal diagnosis for a Chinese pregnant woman with a novel mutation of β thalassemia. Zhonghua Xue Ye Xue Za Zhi.2011; 32:245–248. 21569707

[24] Yang G, Cui JH, Chen S, Si JH. Establishment of a new HBV genotyping method with PCR-RBD and its application. Zhonghua Gan Zang Bing Za Zhi. 2004; 12:677–680. 15623378

[25] Li J, Wang Y, Tian X, Wang P. Analysis of human papillomavirus infection and typing in Shanxi province, Zhonghua Yu Fang Yi Xue Za Zhi.2014;48:192–196. 24844832

[26] Nkhoma ET, Poole C, Vannappagari V, Hall SA, Beutler E. The global prevalence of glucose-6-phosphate dehydrogenase deficiency: a systematic review and meta-analysis. Blood Cells Mol Dis. 2009; 42:267–278. 10.1016/j.bcmd.2008.12.005 19233695

[27] Chan TK, Todd D, Wong CC. Erythrocyte Glucose-6-Phosphate Dehydrogenase Deficiency in Chinese. Br Med J. 1964; 2:102 14147757 10.1136/bmj.2.5401.102 PMC1815978

[28] Xu W, Westwood B, Bartsocas CS, Malcorra-Azpiazu JJ, Indrák K, Beutler E. Glucose-6 phosphate dehydrogenase mutations and haplotypes in various ethnic groups. Blood. 1995; 85: 257–263. 7803800

[29] Tang TK, Liu TH, Tang CJ, Tam KB. Glucose-6-phosphate dehydrogenase (G6PD) mutations associated with F8C/G6PD haplotypes in Chinese. Blood. 1995;85: 3767–3768. 7780161

[30] Beulter E, Westwood B, Kuhl W. Definition of the mutations of G6PD Wayne, G6PD Viangchan, G6PD Jammu, and G6PD ‘LeJeune’. Acta Haematol.1991; 86:179–82. 1805484 10.1159/000204830

